# The Association of Cholesterol Uptake and Synthesis with Histology and Genotype in Cortisol-Producing Adenoma (CPA)

**DOI:** 10.3390/ijms23042174

**Published:** 2022-02-16

**Authors:** Naoki Motomura, Yuto Yamazaki, Daiki Koga, Shogo Harashima, Xin Gao, Yuta Tezuka, Kei Omata, Yoshikiyo Ono, Ryo Morimoto, Fumitoshi Satoh, Yasuhiro Nakamura, Go Eun Kwon, Man Ho Choi, Akihiro Ito, Hironobu Sasano

**Affiliations:** 1 Department of Pathology, Tohoku University Graduate School of Medicine, Sendai 980-8575, Japan; naoki.motomura.p5@dc.tohoku.ac.jp (N.M.); daiki.koga.r4@dc.tohoku.ac.jp (D.K.); sp310daikonistheultimate@gmail.com (S.H.); gaoxin0222@jlu.edu.cn (X.G.); hsasano@patholo2.med.tohoku.ac.jp (H.S.); 2 Division of Clinical Hypertension, Endocrinology and Metabolism, Tohoku University Graduate School of Medicine, Sendai 980-8575, Japan; y.tezuka@med.tohoku.ac.jp (Y.T.); kei.omata.d4@tohoku.ac.jp (K.O.); y-ono@med.tohoku.ac.jp (Y.O.); fsatoh@med.tohoku.ac.jp (F.S.); 3 Division of Nephrology, Endocrinology and Vascular Medicine, Tohoku University Hospital, Sendai 980-8574, Japan; rmorimoto@med.tohoku.ac.jp; 4 Division of Pathology, Faculty of Medicine, Tohoku Medical and Pharmaceutical University, Sendai 981-8558, Japan; yasu-naka@patholo2.med.tohoku.ac.jp; 5 Molecular Recognition Research Center, Korea Institute of Science and Technology, Seoul 02792, Korea; missy2459@gmail.com (G.E.K.); manhokist@gmail.com (M.H.C.); 6 Department of Urology, Tohoku University School of Medicine, Sendai 980-8574, Japan; akihiro.ito.c5@tohoku.ac.jp

**Keywords:** Cushing’s syndrome, cortisol producing adenoma, cholesterol uptake, de novo synthesis, HSL, *PRKACA*, immunohistochemistry, mass spectrometry

## Abstract

Cortisol-producing adenoma (CPA) is composed of clear and compact cells. Clear cells are lipid abundant, and compact ones lipid poor but associated with higher production of steroid hormones. *PRKACA* mutation (*PRKACA* mt) in CPA patients was reported to be associated with more pronounced clinical manifestation of Cushing’s syndrome. In this study, we examined the association of histological features and genotypes with cholesterol uptake receptors and synthetic enzymes in 40 CPA cases, and with the quantitative results obtained by gas chromatography-mass spectrometry (GC-MS) analysis in 33 cases to explore their biological and clinical significance. Both cholesterol uptake receptors and synthetic enzymes were more abundant in compact cells. GC-MS analysis demonstrated that the percentage of compact cells was inversely correlated with the concentrations of cholesterol and cholesterol esters, and positively with the activity of cholesterol biosynthesis from cholesterol esters. In addition, hormone-sensitive lipase (HSL), which catalyzes cholesterol biosynthesis from cholesterol esters, tended to be more abundant in compact cells of *PRKACA* mt CPAs. These results demonstrated that both cholesterol uptake and biosynthesis were more pronounced in compact cells in CPA. In addition, more pronounced HSL expression in compact cells of *PRKACA* mt CPA could contribute to their more pronounced clinical manifestation.

## 1. Introduction

Cortisol is synthesized from cholesterol through the cascades of steroidogenic enzymes including CYP17A1 and CYP11B1 in the zona fasciculata cells of human adrenal cortex. Intracellular free cholesterol is generally provided by three pathways: uptake of plasma cholesterols, metabolism of intracellularly stored cholesterol esters, and de novo biosynthesis [1]. Plasma cholesterol is the major source of cholesterol in steroidogenesis, and both low density lipoprotein receptor (LDL-R) and high density lipoprotein receptor, called scavenger receptor class B member 1 (SR-B1), are the main receptors responsible for plasma cholesterol uptake [1,2,3]. Of particular interest, SR-B1 is also known to mediate the selective uptake of cholesterol esters into the cells in steroidogenesis [3], and its deficiency was reported to result in decreased overall steroid production [4]. Hormone-sensitive lipase (HSL) is responsible for the biosynthesis of cholesterol from cholesterol esters, and both acyl-CoA:cholesterol acyltransferase 1 (ACAT1) and acyl-CoA:cholesterol acyltransferase 2 (ACAT2) catalyze the conversion of free cholesterol into cholesterol esters and also play important roles in maintaining the homeostasis of the intracellular cholesterol levels [1,5]. Several previously reported studies using HSL knockout mice revealed the significance of HSL in steroidogenesis [6,7,8]. In addition, results of in vitro studies using a human adrenocortical carcinoma cell line demonstrated that steroidogenic factor 1, which played a key role in enhancing expression of many essential steroidogenic enzymes, also activated HSL transcription [9,10]. 24-Dehydrocholesterol reductase (DHCR24) is also one of the key enzymes in the de novo cholesterol synthesizing pathway and catalyzes the cholesterol synthesis from acetyl-CoA [1].

Adrenal Cushing’s syndrome (CS) is characterized by autonomous cortisol over-secretion from the adrenal cortex. Cortisol-producing adenoma (CPA) is the most frequent subtype of ACTH-independent CS [11] and is histologically composed of two different cell subtypes: clear and compact tumor cells [12]. Clear cells have abundant lipid droplets, whereas compact cells are small-sized cells with few lipid droplets. We previously demonstrated that the steroidogenic enzyme expression status was higher in compact cells than in clear cells in CPA cases [12]. Both extracellular cholesterol uptake and de novo synthesis were recently reported to be higher in CPA than bilateral adrenal hyperplasia (BAH), indicating that CPA represented cholesterol starved status [13]. However, the association of cholesterol uptake and synthesis with histological features of CPA has remained virtually unknown.

Several previously reported studies revealed the presence of somatic gene mutations of *PRKACA*, *GNAS*, and *CTNNB1* in CPA [14,15,16,17]. In particular, *PRKACA* mutation (*PRKACA* mt) was reported as the most frequently detected genetic alteration among Japanese CPA cases [12,18] and was associated with clinically more pronounced phenotypes of CS such as high plasma cortisol levels and overt CS [19,20,21]. In addition, *PRKACA* mt was reported to directly increase the steroidogenic enzyme expression by activating cAMP-PKA activity, and its gene expression profiles were different from CPAs harboring other mutations [22]. However, the association of cholesterol uptake and synthesis, both of which play pivotal roles in CPA, with the different genotypes above has also remained unexplored.

Therefore, in this study, we comprehensively analyzed the expression status of the receptors and enzymes responsible for cholesterol uptake and biosynthesis in CPAs. We also quantified the free cholesterol and its precursors and firstly examined their associations with tumor cell morphometry as well as genotypes in order to further explore the biological and clinical significance of cholesterol uptake and biosynthesis in CPAs.

## 2. Results

### 2.1. Comparison of Receptors and Enzymes between Clear Cells and Compact Cells

**Figure 1:**
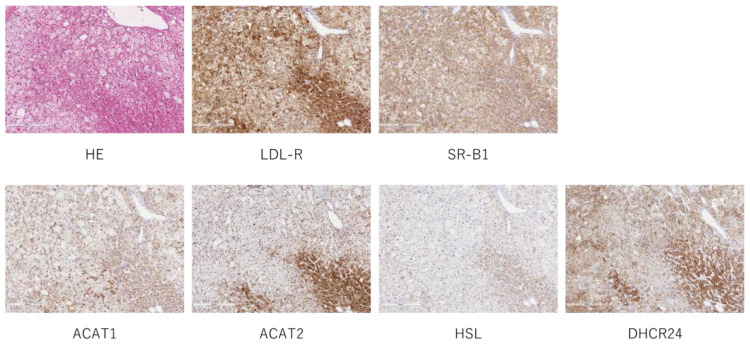
Representative image of H&E staining and IHC. H&E staining and IHC of LDL-R, SR-B1, ACAT1, ACAT2, HSL and DHCR24.

Hematoxylin and eosin (H&E) staining and immunohistochemistry (IHC) of receptors (LDL-R and SR-B1) and enzymes (ACAT1, ACAT2, HSL and DHCR24) were performed in CPAs (Figure 1). Results were semi-quantified and compared between clear and compact tumor cells (Table 1). Membranous immunoreactivity of SR-B1 (*p* = 0.0289), and cytoplasmic immunoreactivity of ACAT1 (*p* = 0.0004), ACAT2 (*p* < 0.0001), HSL (*p* < 0.0001) and DHCR24 (*p* < 0.0001) were significantly higher in compact than in clear tumor cells.

### 2.2. Correlations of Cholesterol Uptake and Biosynthesis with the Morphological Features

The correlation between the percentage of compact cells and concentrations of individual cholesterol and its precursors, their metabolic ratios and immunoreactivity of receptors and enzymes were examined (Table 2). Free cholesterol (ρ = −0.3789, *p* = 0.0299) and cholesterol arachidonate (Chol-A) (ρ = −0.3977, *p* = 0.0219) were both inversely correlated with the percentage of compact tumor cells (Table 2). All of the other cholesterol esters, cholesterol laurate (Chol-L) (ρ = −0.3252, *p* = 0.0648), cholesterol myristate (Chol-M) (ρ = −0.3289, *p* = 0.0617), cholesterol palmitate (Chol-P) (ρ = −0.3229, *p* = 0.0669) and cholesterol oleate, linoleate, and stearate (Chol-Li, O, S) (ρ = −0.3436, *p* = 0.0503) also tended to be inversely correlated with the proportion of compact cells, although the correlation did not reach statistical significance. On the other hand, there were no significant correlations between the concentrations of cholesterol precursors involved in *de novo* pathways and the percentage of compact cells.

The metabolic ratio of free cholesterol to Chol-A (cholesterol/Chol-A), representing the hydrolysis of cholesterol ester, was significantly positively correlated with the percentage of compact tumor cells in CPAs (ρ = 0.4047, *p* = 0.0195) (Table 2). All of the other metabolic ratios corresponding to cholesterol ester metabolism, cholesterol/Chol-L (ρ = 0.2978, *p* = 0.0923), cholesterol/Chol-M (ρ = 0.3282, *p* = 0.0622), cholesterol/Chol-P (ρ = 0.3145, *p* = 0.0747) and cholesterol/Chol-Li, O, S (ρ = 0.3436, *p* = 0.0503) also tended to be positively correlated with the percentage of compact cells. However, among the metabolic ratios corresponding to the *de novo* cholesterol synthesis activity, only cholesterol/7-dehydrocholesterol (7-DHC) tended to be inversely correlated with the percentage of compact tumor cells, but this did not reach statistical significance (ρ = 0.3275, *p* = 0.0628).

ACAT1 (ρ = 0.5893, *p* < 0.0001), ACAT2 (ρ = 0.3713, *p* = 0.0183) and DHCR24 (ρ = 0.4045, *p* = 0.0096) were all significantly positively correlated with the percentage of compact cells (Table 2). Membranous SR-B1 immunoreactivity tended to be inversely correlated with the percentage of compact cells, although the correlation did not reach statistical significance (ρ = 0.2674, *p* = 0.0953).

### 2.3. Differences in Cholesterol Uptake and Biosynthesis between PRKACA mt and Non-PRKACA mt

Differences in tissue concentrations of steroids, metabolic ratios and immunoreactivity of receptors and enzymes were compared between *PRKACA* mt and non-*PRKACA* mt CPA cases (Table 3).

No significant differences in concentrations of steroids examined and metabolic ratios were detected between *PRKACA* mt and non-mt cases. Only HSL immunoreactivity in compact tumor cells tended to be higher in *PRKACA* mt (*p* = 0.0859), although the correlation did not reach statistical significance.

## 3. Discussion

This is the first study to examine the association of cholesterol uptake and synthesis with morphology and genotypes in CPA.

Extracellular cholesterol uptake through LDL-R and SR-B1, and intracellular biosynthesis of free cholesterol by de novo synthesis and cholesterol ester metabolism are well-known sources of free cholesterol [1]. In addition, London E et al. also recently reported that extracellular cholesterol uptake via LDL-R and intracellular cholesterol synthesis through de novo pathways were both increased, and cholesterol efflux was decreased in CPA compared to BAH, which indicated that CPA could correspond to “cholesterol-starved tissues” [13]. On the other hand, the association of cholesterol uptake and synthesis with morphology and genotypes in CPA has remained virtually unknown.

**Figure 2:**
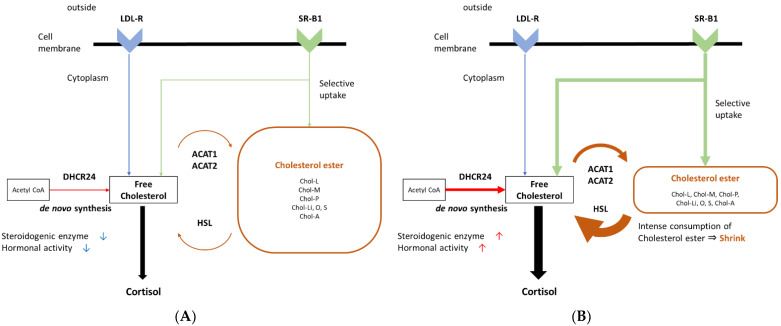
Difference in cholesterol uptake and synthesis depending on morphological features. Differences in cholesterol uptake and metabolism depending on the status of each cell in CPA are illustrated. (**A**): Cholesterol uptake and biosynthesis in relatively cholesterol enriched cells including clear tumor cells. Both cholesterol uptake and synthesis were low, and hormonal activity was also relatively low. (**B**): Cholesterol uptake and synthesis in cholesterol starved cells including compact tumor cells. Both cholesterol uptake and synthesis were increased, and hormonal activity was high. Increased cholesterol synthesis through the cholesterol ester metabolism could contribute to its characteristic shrinked morphology.

In this study, we comprehensively semi-quantified the immunoreactivity of the receptors and enzymes involved in cholesterol uptake or biosynthesis in CPA. SR-B1, HSL and DHCR24 were all significantly higher in compact than clear tumor cells (Table 1). DHCR24 was also significantly positively correlated with the percentage of compact tumor cells (Table 2). In addition, the percentage of compact tumor cells was inversely correlated with the tissue concentrations of free cholesterol and cholesterol esters, and also positively with the activities of the cholesterol biosynthesis through the cholesterol ester metabolism (Table 2). These results all indicated that both plasma cholesterol uptake and intracellular cholesterol biosynthesis including the cholesterol synthesis from the cholesterol ester were activated in compact tumor cells (Figure 2A,B). In addition, lipid droplets in adrenocortical cells were reported to be mainly composed of cholesterol ester [23]. Therefore, the enhanced cholesterol ester metabolism in compact tumor cells detected in our present study was considered to decrease the volume of lipid droplets, subsequently resulting in the relative shrinkage of the cytoplasm compared to clear cells. This could also contribute to the characteristic morphological features of compact tumor cells, i.e., small size with few lipid droplets, and demonstrated that these cells corresponded to cholesterol starved cells. However, further investigations are warranted to clarify the roles of cholesterol ester metabolism in the process of development of these characteristic histological features of tumor cells in CPA.

In addition, we previously reported the associations of steroid hormone synthesis with histology and cellular senescence in CPA [12]. In our previous study, hormonal activity was significantly higher and cell senescence more progressed in compact tumor cells, which suggested that the cell senescence in compact tumor cells was caused by in situ excessive cortisol exposure in local tissue microenvironment [12,24]. In this study, we demonstrated that the supply of free cholesterol was elevated in compact tumor cells (Table 1), which is consistent with the previously reported higher hormonal activity in compact tumor cells and supported the hypothesis that the cell senescence was more pronounced in compact tumor cells by excessive cortisol exposure.

In addition, ACAT1 and ACAT2 were both significantly higher in compact than clear tumor cells (Table 1 and Table 2), suggesting that they were in equilibrium with cholesterol ester metabolism to maintain the homeostasis of intracellular free cholesterol levels [5]. As for the de novo pathway, DHCR24 was higher in compact tumor cells (Table 1) but there were no significant correlations detected between the morphological parameter (the percentage of compact cells) and de novo pathway activities (Table 2). This could indicate the fact that both the enzymes and the sufficient quantity of substrates were required for the metabolism to proceed, and the expression of the synthase did not necessarily directly reflect the progression of metabolism. A previously reported study also demonstrated that the localization of the synthase did not necessarily correspond to that of the metabolites and their precursor [25]. In our present study, the concentrations of steroids involved in the de novo pathway were not necessarily correlated with the percentage of compact tumor cells (Table 2). This could have caused the discrepancy between DHCR24 expression and de novo pathway activity, but it is also important to note that we could not separately analyze the clear and compact tumor cells in our present study using GC-MS. Therefore, further investigations are required to clarify the correlation between histological features and de novo pathway activity, including differences in steroid contents between clear and compact tumor cells of CPA. In addition, the cytoplasmic immunoreactivity of LDL-R and SR-B1 was detected in this study, and we therefore examined the correlations between LDL-R/SR-B1 in the cytoplasm and membrane, respectively (Supplemental Figure S1). Both of them were significantly positively correlated, suggesting the equilibrium between those two above, and cytoplasmic immunoreactivity could represent the cholesterol uptake into the cell.

In our present study, we also examined the correlation between genetic mutations and cholesterol uptake and synthesis. Results demonstrated that HSL in compact tumor cells tended to be higher in the CPA cases harboring *PRKACA* mt than non-*PRKACA* mt cases (Table 3). *PRKACA* mt was reported to increase the expression of steroid hormone synthases [22], and to represent clinically more pronounced phenotypes of CS [19,20,21]. Therefore, the expression of HSL could have been increased following the increased expression of steroidogenic enzymes and demands for the free cholesterol by *PRKACA* mt, and could contribute to more severe phenotypes, e.g., higher serum cortisol levels.

## 4. Materials and Methods

### 4.1. Human Adrenals

A total of 40 cases of CPAs (*PRKACA* mt: *n* = 20, non-*PRKACA* mt: *n* = 20) operated at Tohoku University Hospital, Sendai, Japan from 2015 to 2018 were examined in this study. All of the cases were clinically diagnosed as adrenal CS [26]. Based on the genetic mutations of each case, we retrieved 40 CPAs so that the number of cases of *PRKACA* mt and non-*PRKACA* mt would be equal [12]. The clinical features of 40 cases were summarized in Table 1. For histopathological analysis, 40 cases of 10% formalin fixed paraffin embedded (FFPE) tissues were used. Among these 40 cases, 33 cases (*PRKACA* mt: *n* = 16, non-*PRKACA* mt: *n* = 17) were available as optimal cutting temperature-embedded frozen tissues, and GC-MS analysis was performed in all of these cases.

### 4.2. H&E Staining and IHC

H&E staining and IHC of free cholesterol uptake receptors (LDL-R and SR-B1) and metabolic enzymes (ACAT1, ACAT2, HSL and DHCR24) were performed in FFPE specimens. FFPE tissues were sliced at 3 µm thickness using a microtome (Yamato Kohki Industrial Co., Ltd., Saitama, Japan). IHC protocols were summarized in Supplemental Table S1.

### 4.3. Semi-Quantitative Evaluation of Morphology and Immunoreactivity

All stained tissue sections were digitally scanned by Image Scope AT2 (Leica, Wetzler, Germany). H&E staining was evaluated by HALO TM Area Quantification ver.1.0 (Indica Labs, Corrales, NM, USA) program as previously reported [27]. Tumor cells were tentatively classified into the nucleus and cytoplasm, and they were further classified into compact and clear tumor cells. The percentage of compact cells partly obtained from the historical data [12] were used as the morphological parameters. Cytoplasmic immunoreactivity of all the enzymes examined in this study was evaluated by using HALO TM CytoNuclear ver.1.5 (Indica Labs) program, and semi-quantified [12,27,28,29,30]. Positive cells were tentatively classified into +1 (Weak), +2 (Moderate) and +3 (Strong) according to their immunointensity. The H-Score was subsequently calculated according to the following formula: Σ (Number of individual immunopositive cells × Immunointensity score (+1, +2, +3)/Number of total cells) X 100. Cytoplasmic immunoreactivity of LDL-R and SR-B1 was also semi-quantified according to the same formula, and the correlation with the immunoreactivity in the membrane was examined (Supplemental Figure S1). Immunoreactivity of LDL-R and SR-B1 in the membrane was evaluated by HALO TM Membrane Quantification ver. 1.7 (Indica Labs). PIC-Score was defined and used for the semi-quantification. PIC-Score was calculated according to the following formula: PIC-Score = Percentage of positive cells (Percentage) × Average positive cell membrane optical density (Intensity) × Average positive membrane completeness (Circumference).

### 4.4. Quantitative Metabolic Signatures of Cholesterols Using GC-MS

Free cholesterol, 7 cholesterol esters (Chol-L, Chol-M, Chol-A, Chol-P, Chol-Li, O, S) and 4 cholesterol precursors involved in de novo pathways (desmosterol; Des, 7-DHC, lathosterol; Latho, lanosterol; Lano) were quantitatively analyzed as previously reported [31,32].

Each cholesterol ester metabolism and de novo synthesis activity was analyzed by the corresponding metabolic ratios [33]. The metabolic ratio was calculated according to the following formula: metabolic ratio = the concentration of cholesterol/the concentration of corresponding precursors. Cholesterol/cholesterol esters (Chol-L, Chol-M, Chol-A, Chol-P and Chol-Li, O, S) were used as an index of the cholesterol ester metabolism activity, and cholesterol/cholesterol precursors involved in de novo pathways (Des, 7-DHC, Latho, Lano) were used for the index of de novo synthesis activity.

### 4.5. Statistical Analysis

All the results were statistically analyzed by the software “JMP Pro 16.0”. The correlation between the percentage of compact cells and immunoreactivities of receptors and enzymes, steroids’ concentrations and metabolic ratios were analyzed by the Spearman’s rank correlation coefficient test. The difference in the immunoreactivities of receptors and enzymes between clear cells and compact cells, and the difference in the immunoreactivities of receptors and enzymes, steroids’ concentration, metabolic ratios between *PRKACA* mt and non-*PRKACA* mt were analyzed by Mann–Whitney’s U test. In all statistical analyses, the significance was regarded as *p*-value < 0.05.

## 5. Conclusions

We firstly revealed the association of cholesterol uptake and biosynthesis with the morphology and genotypes of CPA using both semi-quantitative histopathological analysis and GC-MS analysis. In clear tumor cells, in which steroidogenic enzymes were reported low and lipid was abundantly stored, both extracellular cholesterol uptake and intracellular cholesterol biosynthesis were relatively low. On the other hand, in compact tumor cells, where steroidogenic enzymes were relatively abundant and lipid poor, both extracellular cholesterol uptake and intracellular cholesterol biosynthesis were elevated. In particular, the increased activity of the cholesterol biosynthesis from cholesterol esters was demonstrated, which could contribute to the high hormonal activity and the formation of morphological characteristics of compact tumor cells. In addition, HSL in compact tumor cells was more abundant in CPA cases harboring *PRKACA* mt than non mt cases, which could contribute to the development of clinically more severe phenotypes such as higher serum cortisol levels and overt CS.

## Figures and Tables

**Table 1:** Comparison of immunoreactivities of receptors and enzymes between clear cells and compact cells. Immunoreactivity of LDL-R, SR-B1, ACAT1, ACAT2, HSL and DHCR24 in both clear and compact tumor cells was semi-quantified and evaluated. Except for LDL-R, immunoreactivity of all the receptor and enzymes examined in this study were higher in compact than in clear tumor cells. The values of each factor were represented by the median [Range: 1st quartile, 3rd quartile].

Cholesterol Uptake/Synthesis	Clear Cell	Compact Cell	*p*-Value
LDL-R PIC-Score	301.064 [171.6032–409.4358]	358.1922 [208.0366–472.4762]	*p* = 0.2235
SR-B1 PIC-Score	8.0921 [0.0049–101.5824]	80.7402 [2.1292–511.6923]	*p* = 0.0289
ACAT1 H-Score	14.1902 [2.0041–41.7950]	66.8817 [15.3391–126.5957]	*p* = 0.0004
ACAT2 H-Score	68.7927 [23.7838–93.2270]	196.1126 [151.3714–234.4872]	*p* < 0.0001
HSL H-Score	32.4039 [7.5268–71.3349]	139.3665 [84.8874–194.0853]	*p* < 0.0001
DHCR24 H-Score	69.3694 [30.7692–95.6722]	131.619 [99.8727–150.6372]	*p* < 0.0001

**Table 2:** Correlations between the percentage of compact cells and the cholesterol uptake/synthesis. The correlation between the percentage of compact cells and concentrations of individual cholesterol and its precursors, their metabolic ratios and immunoreactivity of receptors and enzymes were examined. Free cholesterol and cholesterol ester (Chol-L, Chol-M, Chol-P, Chol-A, Chol-Li, O, S) concentrations were significantly or tended to be inversely correlated with the percentage of the compact cells. The metabolic ratios corresponding to the activity of cholesterol ester metabolism (cholesterol/Chol-L, cholesterol/Chol-M, cholesterol/Chol-P, cholesterol/Chol-P, cholesterol/Chol-Li, O, S) were significantly or tended to be positively correlated with the percentage of compact cells, and cholesterol/7-DHC tended to be inversely correlated. ACAT1, ACAT2 and DHCR24 were positively correlated with the percentage of compact tumor cells, but SR-B1 tended to be inversely correlated.

Steroids’ Concentration/ Metabolic Ratio /Cholesterol Uptake or Synthesis	Compact Cell (%)
Cholesterol	ρ = −0.3783, *p* = 0.0299
Chol-L	ρ = −0.3252, *p* = 0.0648
Chol-M	ρ = −0.3289, *p* = 0.0617
Chol-P	ρ = −0.3229, *p* = 0.0669
Chol-Li, O, S	ρ = −0.3436, *p* = 0.0503
Chol-A	ρ = −0.3977, *p* = 0.0219
Des	ρ = −0.0267, *p* = 0.8826
7-DHC	ρ = 0.1541, *p* = 0.3919
Latho	ρ = 0.1501, *p* = 0.4045
Lano	ρ = 0.2136, *p* = 0.2327
Cholesterol/Chol-L	ρ = 0.2978, *p* = 0.0923
Cholesterol/Chol-M	ρ = 0.3282, *p* = 0.0622
Cholesterol/Chol-P	ρ = 0.3145, *p* = 0.0747
Cholesterol/Chol-Li, O, S	ρ = 0.3436, *p* = 0.0503
Cholesterol/Chol-A	ρ = 0.4047, *p* = 0.0195
Cholesterol/Des	ρ = −0.2025, *p* = 0.2583
Cholesterol/7-DHC	ρ = −0.3275, *p* = 0.0628
Cholesterol/Latho	ρ = −0.2340, *p* = 0.1900
Cholesterol/Lano	ρ = −0.2774, *p* = 0.1181
LDL-R PIC-Score	ρ = −0.1450, *p* = 0.3719
SR-B1 PIC-Score	ρ = −0.2674, *p* = 0.0953
ACAT1 H-Score	ρ = 0.5893, *p* < 0.0001
ACAT2 H-Score	ρ = 0.3713, *p* = 0.0183
HSL H-Score	ρ = 0.0181, *p* = 0.9117
DHCR24 H-Score	ρ = 0.4045, *p* = 0.0096

Abbreviation; Chol-L (cholesterol laurate), Chol-M (cholesterol myristate), Chol-P, (cholesterol palmitate), Chol-Li, O, S, (cholesterol oleate, linoleate, stearate), Chol-A (cholesterol arachidonate), Des (desmosterol), 7-DHC (7-dehydrocholesterol), Latho (lathosterol), Lano (lanosterol).

**Table 3:** Differences in cholesterol uptake/synthesis between *PRKACA* mt and non-*PRKACA* mt. HSL in compact cells tended to be higher in *PRKACA* mt cases but the difference did not reach statistical significance. The values of each factor were represented by the median [Range: 1st quartile, 3rd quartile].

Steroids’ Concentration/ Metabolic Ratio/ Cholesterol Uptake or Synthesis	*PRKACA* mt	Non-*PRKACA* mt	*p*-Value
Cholesterol	5861.593 [5382.19–7136.4]	5898.944 [4665.091–7147.887]	*p* = 0.7870
Chol-L	46.3632 [29.3517–147.358]	64.4895 [9.1374–180.7776]	*p* = 0.8712
Chol-M	7589.288 [4313.768–20519.77]	10,336.66 [463.5112–25,550.09]	*p* = 0.7322
Chol-P	12,178.57 [6960.808–33,207.79]	14,221.98 [2074.188–28,670.039]	*p* = 0.8430
Chol-Li, O, S	52,515.09 [24,018.31–133,299.5]	60,127.17 [6245.443–13,0951.9]	*p* = 0.8712
Chol-A	158,586.2 [67,639.35–479,610.3]	169,709.2 [12,355.74–283,133.6]	*p* = 0.6525
Des	0.3454 [0.2962–0.3835]	0.3152 [0.2789–0.4438]	*p* = 0.8997
7-DHC	0.4979 [0.4369–0.5973]	0.4975 [0.4432–0.6815]	*p* = 0.5284
Latho	2.2336 [1.4522–3.7237]	3.0636 [1.0444–5.4423]	*p* = 0.8712
Lano	0.4145 [0.2240–1.2060]	0.4596 [0.2714–1.2257]	*p* = 0.6268
Cholesterol/Chol-L	124.5981 [52.8000–187.3307]	92.5434 [42.4202–409.0853]	*p* = 0.8430
Cholesterol/Chol-M	0.7128 [0.3678–1.2558]	0.5590 [0.2862–9.5894]	*p* = 0.6787
Cholesterol/Chol-P	0.4522 [0.2144–0.7773]	0.4714 [0.2330–2.1314]	*p* = 0.7595
Cholesterol/Chol-Li, O, S	0.1026 [0.0583–0.2294]	0.1127 [0.0529–0.7270]	*p* = 0.7870
Cholesterol/Chol-A	0.0342 [0.0163–0.0802]	0.0374 [0.0246–0.3992]	*p* = 0.5052
Cholesterol/Des	19,352.06 [13,583.11–21,680.68]	17,301.79 [13,320.42–20,975.02]	*p* = 0.7322
Cholesterol/7-DHC	12,867.24 [8889.587–15,722.16]	11,032.05 [7518.443–14,785.94]	*p* = 0.3775
Cholesterol/Latho	3216.884 [1521.676–4723.64]	1764.608 [1048.45–6886.364]	*p* = 0.7870
Cholesterol/Lano	17,697.64 [4566.075–28014.47]	14,490.77 [4024.852–24,823.25]	*p* = 0.5284
LDL-R PIC-Score of whole tumor area	255.5031 [204.7156–341.0808]	212.0748 [135.9559–311.1807]	*p* = 0.1556
SR-B1 PIC-Score of whole tumor area	26.7668 [2.4042–131.9024]	8.4648 [0.1653–54.8108]	*p* = 0.1850
ACAT1 H-Score of whole tumor area	35.5899 [6.2073–79.4407]	50.0042 [12.6475–79.9881]	*p* = 0.6949
ACAT2 H-Score of whole tumor area	125.6836 [114.6927–143.0224]	133.8384 [108.0684–145.4952]	*p* = 0.7150
HSL H-Score of whole tumor area	83.0157 [65.9521–109.9652]	61.2836 [22.7286–93.5136]	*p* = 0.1135
DHCR24 H-Score of whole tumor area	118.1096 [109.633–127.5966]	114.9842 [105.6652–124.4034]	*p* = 0.4570
LDL-R PIC-Score of clear cells	261.9615 [177.38–406.2435]	374.8266 [55.4230–417.4899]	*p* = 0.4072
SR-B1 PIC-Score of clear cells	26.4515 [0.1785–68.7130]	0.6045 [2.979 × 10^-5^–145.6982]	*p* = 0.4149
ACAT1 H-Score of clear cells	8.2791 [1.5629–36.5925]	20.8763 [2.0101–54.7858]	*p* = 0.4396
ACAT2 H-Score of clear cells	68.4305 [27.0601–85.8050]	72.2039 [18.6502–99.2369]	*p* = 0.8331
HSL H-Score of clear cells	37.2177 [9.7030–76.4627]	15.2488 [2.2614–68.1975]	*p* = 0.3466
DHCR24 H-Score of clear cells	64.9594 [29.4409–96.4883]	60.7895 [19.7590–95.6722]	*p* = 0.5273
LDL-R PIC-Score of compact cells	273.4003 [146.7106–432.0079]	370.1031 [251.1391–552.4103]	*p* = 0.2503
SR-B1 PIC-Score of compact cells	144.6817 [4.0369–456.7502]	15.6289 [0.3782–301.3014]	*p* = 0.1805
ACAT1 H-Score of compact cells	47.5116 [7.4780–118.3264]	84.5003 [20.8420–140.7282]	*p* = 0.2792
ACAT2 H-Score of compact cells	198.1139 [168.0626–233.9138]	194.5094 [138.6214–237.463]	*p* = 0.5428
HSL H-Score of compact cells	153.2234 [103.4131–199.9192]	110.781 [66.3701–166.0313]	*p* = 0.0859
DHCR24 H-Score of compact cells	131.619 [98.1391–149.6554]	125.5617 [99.8727–155.1823]	*p* = 0.8817

## Data Availability

The data that support the findings of this study are available from the corresponding author upon reasonable request.

## Supplementary Materials

The following supporting information can be downloaded at: https://www.mdpi.com/article/10.3390/ijms23042174/s1.
