# Supplementary material for: The Association of Cholesterol Uptake and Synthesis with Histology and Genotype in Cortisol-Producing Adenoma (CPA)
Source: Int J Mol Sci. 2022 Feb 16;23(4):2174. doi: 10.3390/ijms23042174 (PMC8875534; doi:10.3390/ijms23042174)
Supplement: Supplementary file 1 [file ijms-23-02174-s001.zip › ijms-1560439-supplementary.pdf]

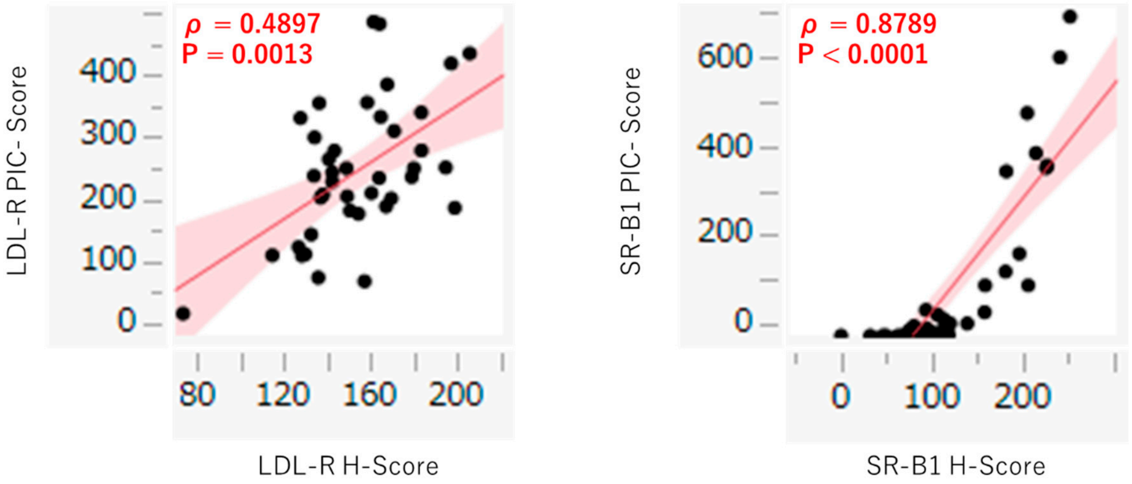

Supplemental Figure S1. Correlations between the immunoreactivity of receptors in the cytoplasm and membrane. The correlations of immunoreactivity of LDL-R or SR-B1 in the cytoplasm and membrane were examined, respectively. Both of them showed significant positive correlations.

Supplemental Table S1. Protocols of IHC. Detailed IHC protocols of LDL-R, SR-B1, ACAT1, ACAT2, HSL and DHCR24.

Antibody	Species	Clone	Antigen retrieval treatment	Dilution ratio	Kit
LDL-R	Rabbit	Monoclonal	AC (pH high)	1:1000	envision FLEX
SR-B1	Rabbit	Monoclonal	AC (pH high)	1:1000	Nichirei
ACAT1	Rabit	Monoclonal	AC (pH=9)	1:100	Nichirei
ACAT2	Mouse	Monoclonal	AC	1:100	Envision +
HSL	Mouse	Monoclonal	AC	1:100	Nichirei
DHCR24	Rabbit	Monoclonal	None	1:500	Nichirei

Abbreviation; AC (autoclave)
